# Evaluation of Tumor Budding and Poorly Defined Clusters as Histological Biomarkers in Squamous Cell Carcinomas of the Vulva †

**DOI:** 10.3390/cancers17101718

**Published:** 2025-05-21

**Authors:** Gilbert Georg Klamminger, Annick Bitterlich, Bashar Haj Hamoud, Erich-Franz Solomayer, Martin Ertz, Laura Schnöder, Bernd Holleczek, Walburgis Brenner, Annette Hasenburg, Mathias Wagner, Meletios P. Nigdelis

**Affiliations:** 1 Department of General and Special Pathology, Saarland University (USAAR) and Saarland University Medical Center (UKS), 66424 Homburg, Germany; 2 Department of Obstetrics and Gynecology, University Medical Center of the Johannes Gutenberg University Mainz, 55131 Mainz, Germany; 3 Department of Gynecology, Obstetrics and Reproductive Medicine, Saarland University Medical Center (UKS), 66424 Homburg, Germany; 4 Saarland University Medical Center for Tumor Diseases (UTS), Saarland University (USAAR), 66424 Homburg, Germany; 5 Saarland Cancer Registry, 66117 Saarbrücken, Germany

**Keywords:** vulvar cancer, tumor budding, poorly defined clusters, histology, tissue-derived biomarker

## Abstract

**Background/Objectives**: Several histopathological risk factors have been examined in vulvar cancer (VC) so far. However, the prognostic relevance of morphological biomarkers such as tumor budding (TB) and poorly defined clusters (PDCs) remains to be determined. **Material and Methods:** We histologically analyzed the formation of peritumoral and intratumoral TB and PDCs in a cohort of 157 patients with VC. We assessed their association with clinico-pathological features and evaluated their prognostic impact in terms of the risk of local recurrence and occurrence of metastasis (Fisher’s exact test) as well as overall survival (Log-rank test). **Results:** We determined a distinct prognostic relevance of peritumoral TB with regard to occurrence of metastasis (Fisher’s exact test; *p* = 0.0415) as well as a significant reduced risk of local recurrence in the group with absent intratumoral TB (Fisher’s exact test; *p* = 0.0004). Furthermore, we showed that patients without peritumoral budding formation had a significant superior prognosis in terms of overall survival (*p* = 0.0366, x^2^ = 4.370). **Conclusions:** This study shows that several new histomorphological biomarkers may serve useful in predicting the clinical course of patients with VC, identifying patients at a lower risk of developing metastases/local recurrence as well as improved overall survival.

## Simple Summary

Although histological features such as intratumoral/peritumoral tumor budding and poorly defined clusters have been previously studied in a variety of solid tumors, very little is yet known about their prognostic relevance in squamous cell carcinomas of the vulva. Therefore, we evaluated the prognostic relevance of the above-mentioned morphological biomarkers in vulvar cancer and indeed determined a superior rate of survival as well as a lower metastasis rate in patients without the formation of tumor buds. We are thus contributing to the establishment of a future research focus on new pathological biomarkers with the ultimate goal of improving the diagnostic and prognostic accuracy in patients with vulvar neoplasia.

## 1. Introduction

Although the self-conception of modern medicine is constantly evolving—methods of molecular biology as well as advanced computer science are regularly integrated in diagnostic processes nowadays—histomorphological tissue diagnostics remain the current gold standard for diagnosing oncological diseases such as vulvar cancer (VC). In squamous cell carcinomas of the vulva (VSCCs), such traditional studies of cell morphology and tumor architecture have identified several histological risk factors (for example, depth of invasion and lymphovascular space invasion) that continue to hold prognostic relevance [1]; their clinical impact is reflected in several international guidelines, and they are therefore routinely identified in pathological reports of vulvar biopsies/vulvectomy specimens [2,3,4]. Although recent research in VC has primarily focused on molecular alterations (HPV status, p53 mutations, involvement of the PI3K/AKT/mTOR pathway) aiming at determining their prognostic potential [5,6,7,8], only a few studies have examined the prognostic relevance of tissue-derived histological biomarkers such as tumor budding or spindle cell morphology [9,10,11], despite their potential global accessibility and cost-effectiveness. However, systematic approaches for a standardized assessment of several noteworthy histological biomarkers, already well established in various alternative solid tumor entities [12,13], are currently lacking in VC.

In our study, we therefore assessed intratumoral and peritumoral tumor bud formation (tumor budding, TB) as well as poorly defined clusters (PDCs) in VSCCs. We examined their association with traditional histopathological features (e.g., inguinal lymph node metastasis and perineural invasion) as well as their prognostic potential with regard to overall survival of the patients and risk of tumor recurrence and metastasis, respectively.

## 2. Materials and Methods

A total of 157 patients with histomorphologically diagnosed VSCC at the Institute of Pathology, University of Saarland, Germany, between 2007 and 2023 were identified as eligible for this study. A priori defined inclusion and exclusion criteria, listed in Table S1, resulted in the exclusion of a total of 9 patients. Pathological histomorphological diagnostics were performed according to good clinical practice and current diagnostic standards (hematoxylin and eosin (H&E) staining, immunohistochemistry, and in situ hybridization, if applicable) in alignment with relevant national and European guidelines [2,3]. Chart reviews were performed. Baseline clinical patient data as well as traditional histopathological parameters were subsequently collected including age, tumor stage, HPV-association (defined by “block-type” immunohistochemical p16 staining pattern; Figure S1), inguinal lymph node involvement (N-stage), lymphovascular space invasion, vascular invasion, perineural infiltration, and infiltration depth. For study purposes, all pathological data were re-staged according to the actual 8th edition of the TNM classification of malignant tumors from 2018. Data on the follow-up of the patients with regard to overall survival, local recurrence status, and occurrence of metastasis were provided and compiled from two sources: the ‘Medical Center for Tumor Diseases’ at Saarland University and the statewide-operating ‘Saarland Cancer Registry’. The study was approved by the Ethics Committee of Saarland, Germany (study identification number 249/23).

**Figure 1:**
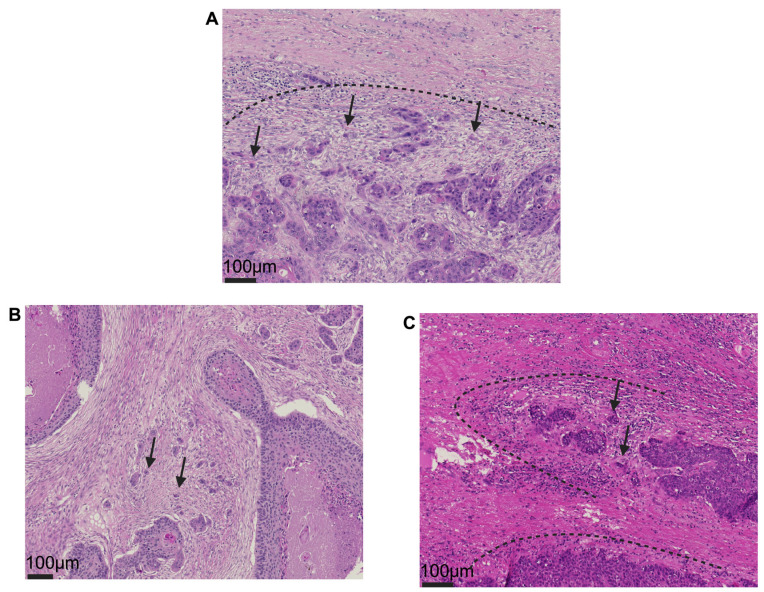
(**A**): Peritumoral tumor budding formation defined as detaching tumor clusters consisting of ≤4 epithelial neoplastic cells at the invasive tumor front (the black arrow indicates representative individual buds). (**B**): Budding formation within the tumor center (so-called intratumoral budding); the black arrow sign marks individual buds exemplary. (**C**): Poorly defined clusters are found within the peritumoral stroma adjacent to the invasive front and are defined as infiltrating formations ≥ 5 neoplastic cells (black arrow sign). The dash line visualizes the invasive tumor front (**A**,**C**). All specimens shown are stained with hematoxylin and eosin (H&E).

Histomorphological tumor/HE slide re-evaluation of included cases was conducted together using a standard multi-head light microscope (GGK, MN, MW), and additional four histomorphological parameters were acquired using the following standardized protocol: Considering a morphological manifestation of the epithelial–mesenchymal transition (EMT), TB is defined as the presence of isolated single cells or small cell clusters, comprising up to four infiltrating neoplastic epithelial cells as stated by the International Tumor Budding Consensus Conference (ITBCC) [14]. A single ‘hotspot’ area, defined by the maximal extent or highest intensity of budding formation at the invasive tumor–stroma front (peritumoral) and within the tumor itself (intratumoral), was identified through the manual screening of at least 10 distinct fields using a ×20 objective. Tumor buds were then counted per one high-power field (HPF; ×40) in the intratumoral hotspot area (*intratumoral TB*) as well as at the invasive front (conventional *peritumoral TB*). For consecutive analysis, the cohort was split separately for each location into a TB-positive group with ≥1 TB and a TB-negative group without any apparent budding formations. Differing from TB, PDCs are defined as clusters of ≥5 neoplastic cells surrounded by stromal tissue components, and all clusters identifiable within the microscopic area of a ×20 objective lens were counted; cases were classified either as PDC-positive (≥1 PDC) or PDC-negative (no PDC observable) [15]. Figure 1 exemplary visualizes typical phenotypic aspects of all aforementioned variables.

Descriptive statistics (GraphPad, Boston, Version 10.4.2, MA 02110, USA) were calculated for clinico-pathological data as well as overall survival, local recurrence, and risk of metastasis with regard to our obtained histomorphological biomarkers of interest. Initial Spearman Rho analysis (with histomorphological biomarkers as discrete variables) was performed to identify correlations, and *p* < 0.05 served as our threshold for statistical significance (approximate *p* value for nonparametric correlation). Differences between our subsequently defined groups (with histomorphological biomarkers now defined as categorical/binary variables) were assessed using Fisher’s exact test. For comparison involving continuous variables (e.g., infiltration depth) the Mann–Whitney test was employed. The Log-rank (Mantel–Cox) test was employed to evaluate the prognostic relevance on overall survival; α < 0.05 was set as threshold defining a test result’s significance.

## 3. Results

### 3.1. Clinico-Pathologic Characteristics

Key characteristics of our study cohort (n = 157 VSCC) are depicted in Table S2. Most cases showed absence of intratumoral budding (n = 96; 61.2%), but instead showed peritumoral budding (n = 120; 76.4%), as well as the presence of PDCs (n = 123; 78.3%). The median age of our cohort was 66 years (interquartile range (IQR): 53–79 years) and 33 (21.0%) patients showed positive inguinal lymph node involvement. In accordance with the 2020 WHO Classification of Female Genital Tumors, tumor entities were defined as either HPV-associated (n = 25; 15.9%), HPV-independent (n = 54; 34.4%), or squamous cell carcinoma of the vulva NOS (not otherwise specified; n = 78; 49.7%). At a median follow-up of 34 months (IQR: 14–72.5), a total of 14 (8.9%) patients developed distant metastases and 31 (19.7%) patients were diagnosed with local vulvar recurrence; distinct classic risk factors associated with recurrence in our study cohort are shown in Table S3.

### 3.2. The Prognostic Relevance of TB and PDCs in VSCCs

**Figure 2:**
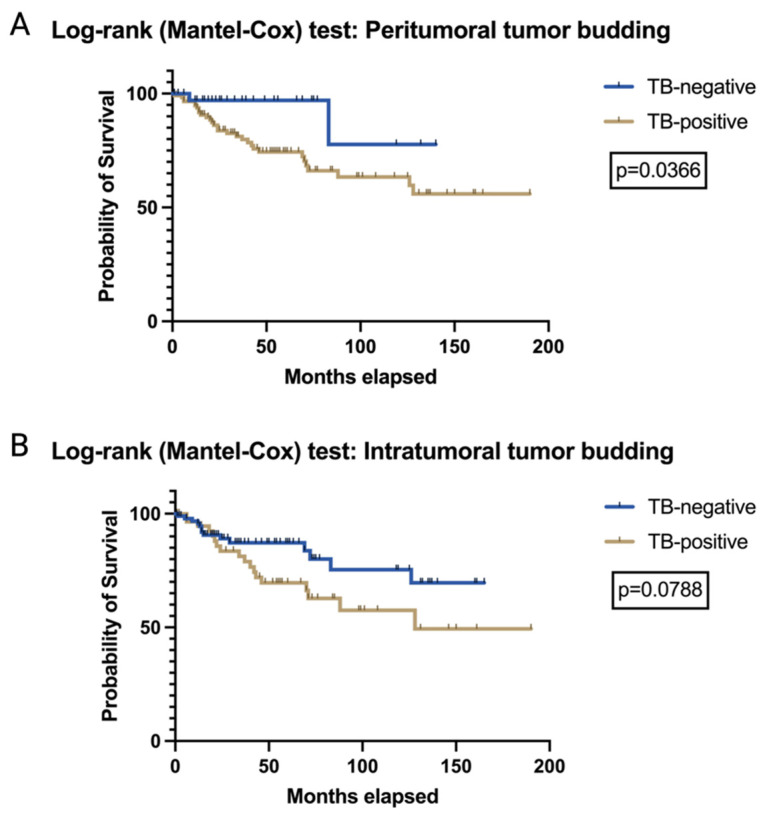
Survival of VSCC patients according to peritumoral (**A**) and intratumoral (**B**) budding formation, demonstrating the prognostic value of peritumoral TB formation in terms of overall survival (Log-rank test; *p* = 0.0366; x^2^ = 4.370).

Evaluating associations of peritumoral/intratumoral TB formations with traditional clinicopathological parameters employing an initial correlation analysis revealed a significant correlation between the total number of peritumoral buds and tumor stage (r = 0.4137; *p* < 0.0001), depth of infiltration (r = 0.4352; *p* < 0.0001), perineural infiltration (r = 0.1840; *p* = 0.0210), as well as the extent of inguinal lymph node metastasis (r = 0.1618; *p* = 0.0429). The number of intratumoral buds instead was positively correlated with tumor stage (r = 0.2981; *p* = 0.0002), depth of infiltration (r = 0.4186; *p* = <0.0001), vascular space infiltration (r = 0.1874; *p* = 0.0188), and extent of inguinal lymph node affection (r = 0.2084; *p* = 0.0088). Tables S4 and S5 list detailed information of our correlation analysis. Splitting our entire study collective in a peritumoral TB-positive/TB-negative cohort, we determined a significant difference between these groups regarding occurrence of metastasis (Fisher’s exact test; *p* = 0.0415); however, the risk of developing a local recurrence did not show a relevant difference between the two groups put to test (Fisher’s exact test; *p* = 0.1573). Subsequent Log-rank testing demonstrated significantly superior overall survival rates for our peritumoral TB-negative cohort (Log-rank test; *p* = 0.0366; x^2^ = 4.370), see Figure 2A for the corresponding Kaplan–Meier curve. Additionally, a split into an intratumoral TB-positive and a TB-negative cohort with subsequent group comparison showed significant differences in terms of metastasis occurrence (Fisher’s exact test; *p* = 0.0486) as well as local recurrence (Fisher’s exact test; *p* = 0.0004) between both groups; however, its prognostic value is not reflected with regard to overall survival (Log-rank test, *p* = 0.0788; x^2^ = 3.089; Figure 2B).

Although PDCs showed positive correlation with certain traditional histopathological parameters (PDC: association with tumor stage (r = 0.4126; *p* ≤ 0.0001) and infiltration depth (r = 0.4134; *p* ≤ 0.0001); refer to Table S6) these associations are not reflected in statistically significant differences between a PDC-positive and a PDC-negative cohort regarding the occurrence of metastasis (Fisher’s exact test; *p* = 0.3054) or local tumor recurrence (Fisher’s exact test; *p* = 0.4746). Thus, no differences in terms of overall survival between the groups analyzed could be observed (PDC-positive vs. PDC-negative: Log-rank test; *p* = 0.0557, x^2^ = 3.662) as shown in Figure S2. A final comprehensive summary of all group comparisons regarding recurrence and metastasis is presented in Table S7.

### 3.3. Association of TB and PDCs with HPV Status

Evaluating the association of our histomorphological biomarker groups with pathophysiological tumorigenesis, viz. HPV-association/HPV-independence, we did not determine any statistically relevant connotation of HPV status and respective histology (Fisher’s exact test: intratumoral TB: *p* = 0.2843; peritumoral TB: *p* > 0.9999; PDC: *p* = 0.3845), as shown in Table S8.

## 4. Discussion

In this study, we analyzed the prognostic value of the tissue-derived histological biomarkers intra/peritumoral TB as well as PDCs in HPV-associated and HPV-independent VSCCs. We demonstrated their various associations with traditional clinico-pathological parameters, which initially suggest their possible integration into an established diagnostic system consisting of pathological factors useful for risk stratification or clinical decision making. Subsequent analysis revealed that determination of intra- and peritumoral budding formation may serve useful in identifying patients at higher risk of metastasis and in the detection of intratumoral TB for assessing risk of local recurrence. Furthermore, our data revealed that patients without signs of peritumoral budding formation show significantly superior overall survival rates in comparison to patients with tumors presenting tumor buds at the peritumoral invasive tumor front. That said, our study further demonstrated that PDCs may not serve as a reliable beneficial prognostic marker in VC.

Although the concept of peritumoral tumor buds—detaching tumor formations that reflect a distinct interaction between neoplasia and surrounding peritumoral soft tissue and thereby resembling epithelial–mesenchymal transformation [16,17]—is a well-studied and established prognostic parameter across several tumor entities such as head and neck cancer and adenocarcinomas of the colon/rectum [12,14], relatively little evidence exists concerning gynecologic neoplasms. Although a recent meta-analysis by Ailia et al. highlights its potential as a prognostic marker in cervical and endometrial cancer, their conclusion remains based on a small number of studies conducted, further accompanied by a lack of standardization between them as the authors precisely point out [13]. With respect to VC, the group of Zare et al. broke new ground when evaluating peritumoral TB in 82 cases of VC for the first time, showing not only its impact on overall survival but also its aim at deciphering its associations with HPV status as well as p53 mutational status [9]—a finding especially true for the field of head and neck cancer not only for research groups such as Stögbauer et al. but also our own studies, which showed a clear association of higher budding formation with a negative HPV status [18,19]. Interestingly, the second research group who examined budding formation in VC in 2023 when evaluating distinct molecular subtypes of VSCC, led by Dongre et al., also postulated a distinct budding in relation to the underlying p16 status [11]. Contrary to such findings, our results do not support a statistically significant correlation of peritumoral budding formation and HPV status. While differences in HPV detection methods (such as p16 immunohistochemistry or RNA in situ hybridization) are unlikely to be the primary cause of the divergent findings, variations in the assessment of TB—such as evaluating buds across 10 high-power fields, a single field at 20× magnification, or, as demonstrated in our study, a single field using a 40× objective—may have contributed to the discrepancy. Additionally, differences in statistical interpretation, such as the use of a budding cut-off of ≥5 tumor buds or a three-tiered classification system (no/low/high budding) versus the ≥1 TB threshold applied in our study, could have influenced the results and led to the respective association with HPV-related tumorigenesis [9,11].

Regarding the detection mode of histological biomarkers, it was Zare et al. who analyzed TB on H&E stainings, as with the present study. However, they chose an alternative approach of evaluation by summing up all counted bud formations in a total of 10 high-power fields and thus dividing their cohort into a three-tier system (no budding/low budding was defined as 1–14 buds/high budding was defined as 15 buds). While the latter approach has proven solid in research efforts of various cancer entities [20,21,22], our proposed approach, in contrast, analyzes all buds per one HPF, aiming to reproduce the most common counting approach of TB in squamous cell carcinomas of the head and neck region, as well as in VC [12]. Since standardized cut-off values are not yet established, we aimed for a dichotomic cut-off (presence/absence) of tumor bud formation [12,23], which—from a biological point of view—ideally represents the favorable state of total absence of mesenchymal transformation of neoplastic cells. In contrast, intratumoral TB, initially described in 1989 [24], as well as PDC are less well-studied phenomena that have not yet established their role in routine pathological reporting [14]. Nevertheless, the prognostic significance of novel histological biomarkers has been repeatedly postulated inter alia in cervical cancer, endometrial cancer, ovarian cancer, breast cancer, external auditory canal carcinoma, and colorectal cancer [25,26,27,28,29,30,31]. To the best of our knowledge, neither PDC nor intratumoral budding formation have been evaluated in VSCC so far.

Hence, one noticeable strength of our study lies in its innovative approach—by evaluating new potential histomorphological biomarkers in a systematic way and in accordance with previous studies. By analyzing surgical material of a large cohort of 157 cases, it furthermore contributes to addressing the current research gap in rare diseases [32]. Moreover, all our histological biomarkers put to test are relatively easy applicable using only standard H&E staining, making them a suitable global tool for cancer research and diagnostics. In contrast to more advanced and sophisticated diagnostic approaches such as DNA methylation profiling [33], our approach can be performed promptly. Furthermore, usage of the statewide-operating cancer registry as an additional source of information allowed for usage of the best possible follow-up information for survival estimation. Last but not least, the approach proposed in our study differs distinctly from traditional prognostic scoring systems, which primarily rely on anatomical classifications such as the TNM or FIGO system. Unlike these staging methods, which emphasize the extent of tumor invasion, our parameters put to test are intended to capture the intrinsic biological behavior of the tumor, irrespective of its anatomical location. Nevertheless, as our findings revealed a positive correlation between TB as well as PDCs and tumor stage, it is likely that these factors are interrelated. Therefore, future research should explore the potential benefits of integrating histopathological biomarkers like TB and PDCs with conventional staging systems, aiming at enhanced prognostic accuracy and improved individualized risk stratification.

One limitation of our study is the lack of linkage of our histomorphological findings with additional molecular pathological and immunological data, such as underlying p53 mutation or PD-L1 status. Another general limitation to acknowledge is that, although inguinal lymph node metastases are typically assessed histologically in clinical routine, a surgical removal of clinically negative lymph nodes is not always performed, well in line with established clinical guidelines. During the study period, lymph node status was clinically assessed for all tumors, either in accordance with current European guidelines or, in earlier years, through more invasive (surgical) procedures. Specifically, in patients with tumors exhibiting a depth of invasion of ≤1 mm and no clinical suspicion of lymph node involvement, clinical assessment of the inguinal lymph nodes alone is considered adequate. In all other cases, either unilateral/bilateral inguinofemoral lymphadenectomy or sentinel lymph node (SLN) biopsy and consecutive pathological workup of the specimens was performed. That said, a detailed analysis of purely clinical parameters—such as the specific type of surgical procedure (e.g., anterior or posterior vulvectomy, wide local excision) or the precise anatomical sites of vulvar recurrence—was beyond the scope of this study.

Since no reliable histological grading system for VSCCs has been established so far [34], our results may provide the basis to include new and non-traditional histological biomarkers in future studies, aiming at achieving a sufficient morphological grading that reliably identifies patients at high risk. That said, the prognostic potential of all variables analyzed here as well as their integrated usage in novel risk models (grading systems) should be confirmed in further independent studies. Special attention should be paid to the standardization of parameter assessment (counting approaches, staining techniques, cut-off values) in order to avoid redundancies and to ensure comparability of individual studies. In addition, the relationship of histomorphological tumor aspects and clinical aspects such as response to neoadjuvant chemotherapy or adjuvant checkpoint inhibitor-based treatment should be assessed [35,36,37,38]. Last but not least, the potential role of such histomorphological biomarkers in shaping treatment strategies and, therefore, their assessment in vulvar biopsy material, needs to be resolved.

## 5. Conclusions

Our study demonstrated that new histomorphological biomarkers such as TB and PDCs may be useful as diagnostic tools with prognostic potential in VSCCs. We identified patients at lower risk of developing metastasis and recurrence based on the assessment of tumor bud formation and determined a better prognosis with respect to overall survival in case of absent peritumoral budding.

## Data Availability

Please contact the corresponding author for individual solutions.

## Supplementary Materials

The following supporting information can be downloaded at: https://www.mdpi.com/article/10.3390/cancers17101718/s1, Figure S1: p16-staining patterns; Figure S2: Log-Rank test:PDC; Table S1: Depiction of a priori defined inclusion/exclusion criteria; Table S2: Depiction of clinico-pathological key characteristics of our entire study cohort; Table S3: Risk of local recurrence in accordance to traditional risk factors; Table S4: Spearman correlation analysis of the amount of peritumoral TB with selected traditional clinicopathological parameters; Table S5: Spearman correlation analysis of the amount of intratumoral TB with selected traditional clinicopathological parameters; Table S6: Spearman correlation analysis of the amount of PDC with selected traditional clinicopathological parameters; Table S7: Summary of group comparisons with respect to recurrence and metastasis; Table S8: Display of the association of our histomorphological biomarker groups put to test with pathophysiological tumorigenesis.
