# Supplementary material for: Evaluation of Tumor Budding and Poorly Defined Clusters as Histological Biomarkers in Squamous Cell Carcinomas of the Vulva
Source: Cancers (Basel). 2025 May 21;17(10):1718. doi: 10.3390/cancers17101718 (PMC12109898; doi:10.3390/cancers17101718)
Supplement: Supplementary file 1 [file cancers-17-01718-s001.zip › cancers-3586488-supplementary.pdf]

SUPPLEMENTARY MATERIAL

Title: Evaluation of Tumor Budding and Poorly Defined Clusters as Histological Biomarkers in Squamous Cell Carcinoma of the Vulva

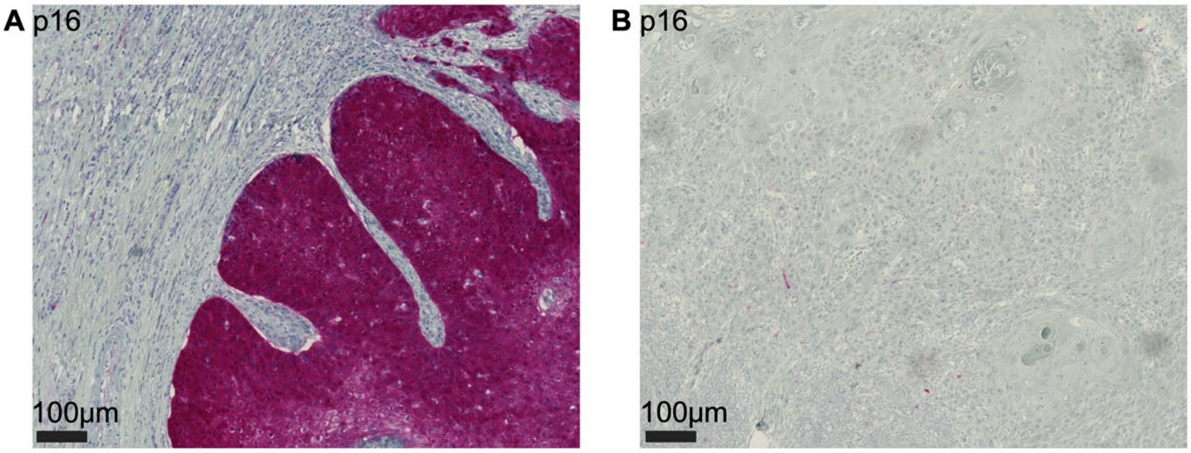

Figure S1. HPV-association is defined by the so-called “block-type” immunohistochemical p16 staining pattern of >20 neighboring positive tumor cells (**A**). Simple cytoplasmatic or faint nuclear stainings are not considered (**B**, HPV-independent tumor).

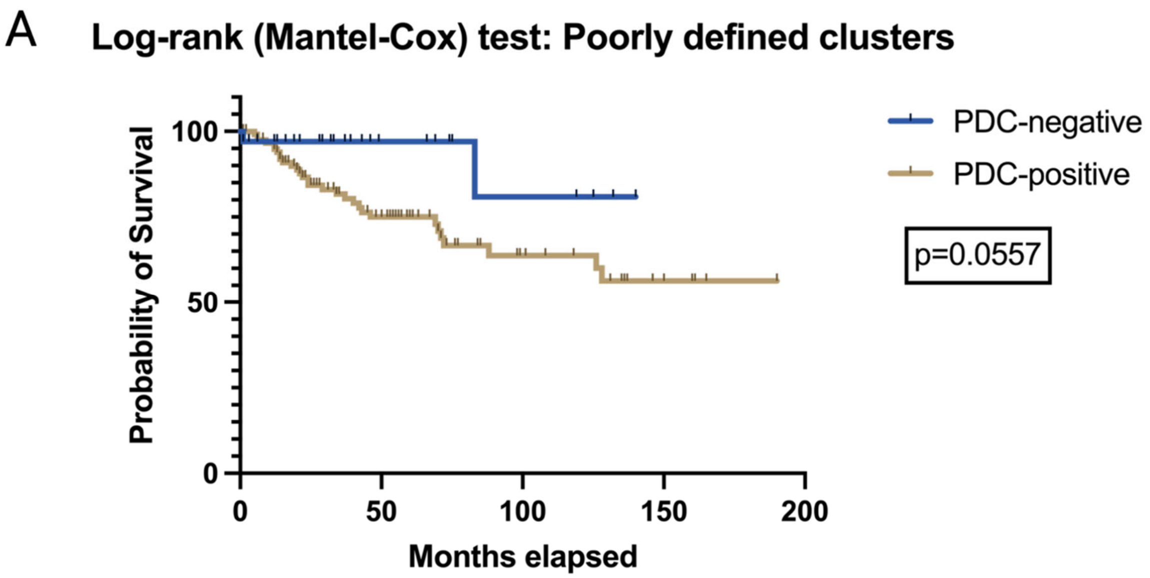

Figure S2. Groups separated in accordance to their respective PDC-status (**A**) did not differ significantly in their overall survival rates (PDC: Log-rank test; p=0.0557, x^2=3.662).

Inclusion criteria	Exclusion criteria
Squamous cell carcinoma of the vulva	High grade squamous intraepithelial (HSIL) lesions of the vulva
Vulvectomy/wide excision specimen available for pathological diagnosis	Vulvar neoplasms other than squamous cell carcinomas (melanomas, adenocarcinomas)
Availability of basic oncological follow up data (occurrence of metastasis, recurrence; overall survival)	Recurrent vulvar lesions (defined as rpT according to the TNM classification)
-	palliative surgery, primary (palliative) radiotherapy

Table S1. Depiction of a priori defined inclusion/exclusion criteria.

clinicopathological data / variables	N=157
Age, years	66 (median), (IQR: 53 - 79)
Histology: HPV-associated	25 (15.9%)
Histology: HPV-independent	54 (34.4%)
Histology: not otherwise specified (nos)	78 (49.7%)
T1a	30 (19.1%)
T1b	108 (68.8%)
T2	19 (12.1%)
N0	124 (79.0%)
positive inguinal lymph node affection (Nmic/N1a to N2c)	33 (21.0%)
L0	132 (84.1%)
L1	25 (15.9%)
V0	146 (93.0%)
V1	11 (7.0%)
Pn0	144 (91.7%)
Pn1	13 (8.3%)
infiltration depth (in cm)	0.7134 (mean), 0.8120 (std. deviation)
intratumoral TB: TB-positive	61 (38.8%)
intratumoral TB: TB-negative	96 (61.2%)
peritumoral TB: TB-positive	120 (76.4%)
peritumoral TB: TB-negative	37 (23.6%)
PDC-positive	123 (78.3%)
PDC-negative	34 (21.7%)

Table S2. Depiction of clinico-pathological key characteristics of our entire study cohort. IQR: interquartile range. PDC: poorly defined clusters. TB: tumor budding.

**clinical parameter of interest with regard to risk of local recurrence**	**p-value**	**statistical analysis**
presence of inguinal lymph node metastasis	**0.0462**	Fisher's exact test
T-stage	0.4742	Fisher's exact test
lymphovascular space invasion	0.0508	Fisher's exact test
infiltration depth (cm)	**<0.0001**	Mann–Whitney test

Table S3 shows the risk of a development of local recurrence in accordance to the traditional classic risk factors.

**Spearman correlation: Peritumoral tumor budding** formation in association to -	**r**	95% confidence interval	**p value (two-tailed)**
tumor stage	0.4137	0.2705 to 0.5389	**<0.0001**
infiltration depth	0.4352	0.2947 to 0.5573	**<0.0001**
vascular space invasion	0.1347	-0.0271 to 0.2896	0.0926
perineural infiltration	0.1840	0.0235 to 0.3353	**0.0210**
lympho-vascular invasion	0.1338	-0.0280 to 0.2887	0.0949
amount of inguinal lymph node affection	0.1618	0.0006 to 0.3148	**0.0429**
age	-0.0426	-0.2024 to 0.1194	0.5962

Table S4. Spearman correlation analysis of the amount of peritumoral tumor budding (TB) with selected traditional clinicopathological parameters.

**Spearman correlation: Intratumoral tumor budding** formation in association to -	**r**	95% confidence interval	**p value (two-tailed)**
tumor stage	0.2918	0.1371 to 0.4327	**0.0002**
infiltration depth	0.4186	0.2761 to 0.5432	**<0.0001**
vascular space invasion	0.1874	0.0270 to 0.3383	**0.0188**
perineural infiltration	0.0649	-0.0973 to 0.2238	0.4191
lympho-vascular invasion	0.1575	0,0038 to 0,3108	0.0489
amount of inguinal lymph node affection	0.2084	0.0489 to 0.3576	**0.0088**
age	0.0220	-0.1397 to 0.1826	0.7844

Table S5. Spearman correlation analysis of the amount of intratumoral tumor budding (TB) with selected traditional clinicopathological parameters.

**Spearman correlation: Poorly defined cluster** formation in association to -	**r**	95% confidence interval	**p value (two-tailed)**
tumor stage	0.4126	0.2693 to 0.5380	**<0.0001**
infiltration depth	0.4134	0.2702 to 0.5387	**<0.0001**
vascular space invasion	0.1092	-0.0529 to 0.2657	0.1734
perineural infiltration	0.0852	-0.0770 to 0.2431	0.2886
lympho-vascular invasion	0.1220	-0.0400 to 0.2777	0.1280
amount of inguinal lymph node affection	0.1314	-0.0304 to 0.2865	0.1009
age	-0.0338	-0.1940 to 0.1281	0.6740

Table S6. Spearman correlation analysis of the amount of poorly differentiated clusters (PDCs) with selected traditional clinicopathological parameters.

**variable of interest**	**group comparison**	**p-value**	**statistical analysis**
Peritumoral TB: occurrence of metastasis	TB-positive vs. TB-negative	**p=0.0415**	Fisher's exact test
Peritumoral TB: occurrence of recurrence	TB-positive vs. TB-negative	p=0.1573	Fisher's exact test
Intratumoral TB: occurrence of metastasis	TB-positive vs. TB-negative	**p=0.0486**	Fisher's exact test
Intratumoral TB: occurrence of recurrence	TB-positive vs. TB-negative	**p=0.0004**	Fisher's exact test
PDC: occurrence of metastasis	PDC-positive vs. PDC-negative	p=0.3054	Fisher's exact test
PDC: occurrence of recurrence	PDC-positive vs. PDC-negative	p=0.4746	Fisher's exact test

Table S7. Summary of group comparisons with respect to recurrence and metastasis. PDC: poorly defined clusters. TB: tumor budding.

clinical parameter of interest with regard to HPV-status	p-value	statistical analysis
Intratumoral TB	p=0.2843	Fisher's exact test
Peritumoral TB	p>0.9999	Fisher's exact test
PDC	p=0.3845	Fisher's exact test

Table S8. Display of the association of our histomorphological biomarker groups put to test with pathophysiological tumorigenesis.
